# Performance of ChatGPT and Gemini Compared with Emergency Physicians in NSTEMI Cases: A Prospective Cross-sectional Study

**DOI:** 10.34172/aim.35274

**Published:** 2025-12-01

**Authors:** Mustafa Yorgancıoğlu, Ekim Saglam Gurmen

**Affiliations:** ^1^Emergency Department, Torbalı State Hospital, İzmir, Turkey; ^2^Emergency Department, School of Medicine, Manisa Celal Bayar University, Manisa, Turkey

**Keywords:** Artificial intelligence, Clinical decision support, Emergency medicine, Large language models, Non-ST elevation myocardial infarction

## Abstract

**Background:**

Diagnosing non-ST elevation myocardial infarction (NSTEMI) in busy emergency departments is challenging. Artificial intelligence (AI) systems, particularly large language models (LLMs), offer potential as clinical decision support tools. This study aimed to evaluate the reliability of ChatGPT and Gemini in NSTEMI cases by comparing their responses to multiple-choice questions with those of emergency physicians.

**Methods:**

This prospective, cross-sectional study was conducted via an online survey among 1,106 emergency physicians in Turkey. The survey included ten NSTEMI-related multiple-choice questions based on the 2023 European Society of Cardiology guidelines. The same questions were presented to ChatGPT 4.0 and Gemini 2.5, queried using identical standardized prompts (temperature=0, no web access) on April 20, 2025. Statistical analyses were performed using SPSS 26.0.

**Results:**

AI models significantly outperformed physicians, correctly answering nine of ten questions versus the physicians’ mean of 7.62±1.32 (*P*<0.001). Effect sizes indicated a very large difference for less experienced physicians and a moderate difference for specialists. Performance improved with experience, yet AI exceeded even the most experienced physicians. Participants from training and research hospitals scored higher than those from state hospitals.

**Conclusion:**

ChatGPT and Gemini demonstrated superior performance over emergency physicians in NSTEMI clinical questions, highlighting AI’s potential to enhance medical education, clinical decision support, and patient care. These findings, however, are limited by the non-proctored online setting and absence of real clinical context. Future research should focus on optimizing AI-clinician collaboration for safe and effective integration.

## Introduction

 Acute coronary syndromes (ACSs) remain a major contributor to global cardiovascular mortality and present frequently in emergency care settings.^1^ Non-ST elevation myocardial infarction (NSTEMI) constitutes a significant proportion among the subtypes of ACSs; however, its diagnostic process is inherently more challenging and variable compared to ST-elevation infarction. A reliable diagnosis typically integrates a patient’s history, electrocardiographic (ECG) interpretation, and cardiac biomarker analysis. Despite these requirements, the high patient volume, time constraints, and overcrowding in emergency departments complicate the diagnostic process.

 Artificial intelligence (AI) increasingly contributes to decision-making in diverse domains, ranging from education to clinical care. In particular, AI systems based on large language models (LLMs), such as ChatGPT and Gemini, may interpret clinical situations and generate plausible suggestions based on natural language input.^2,3^ The potential of AI in medical education to improve learning and assessment methods is increasingly being recognized. Its ability to process and analyze large datasets offers a unique advantage in creating a more dynamic and interactive learning environment.^4^

 Comparative studies between AI-generated answers and physicians’ performance on multiple-choice assessments have gained prominence in recent years. The findings provide important insight into how AI and physicians may complement each other in medical education, as well as into the respective strengths and limitations of both.^5^

 Although successful applications of AI have been reported in cardiovascular fields such as ECG interpretation, risk scoring, and diagnostic processes, studies comparing its diagnostic accuracy with physician performance in more complex clinical conditions like NSTEMI remain limited.^6^ Therefore, this study aimed to assess the diagnostic accuracy of ChatGPT and Gemini in NSTEMI-related clinical scenarios by comparing their responses to those of emergency physicians on multiple-choice questions derived from current ESC guidelines. The primary outcome was the proportion of correct responses by each group.

## Materials and Methods

 This prospective, cross-sectional study was conducted using an online survey between February 15 and April 27, 2025. The target population included emergency physicians across Turkey. A total of 1,106 emergency physicians voluntarily participated. Individuals aged 18 and above were included, while incomplete forms, responses from non-physician healthcare workers, and those not currently working in emergency departments were excluded. The study was designed and reported in accordance with the STROBE and STARD guidelines for cross-sectional diagnostic accuracy studies, and aligned with TRIPOD-AI recommendations for reporting AI-based diagnostic model evaluations.

**Figure 1:**
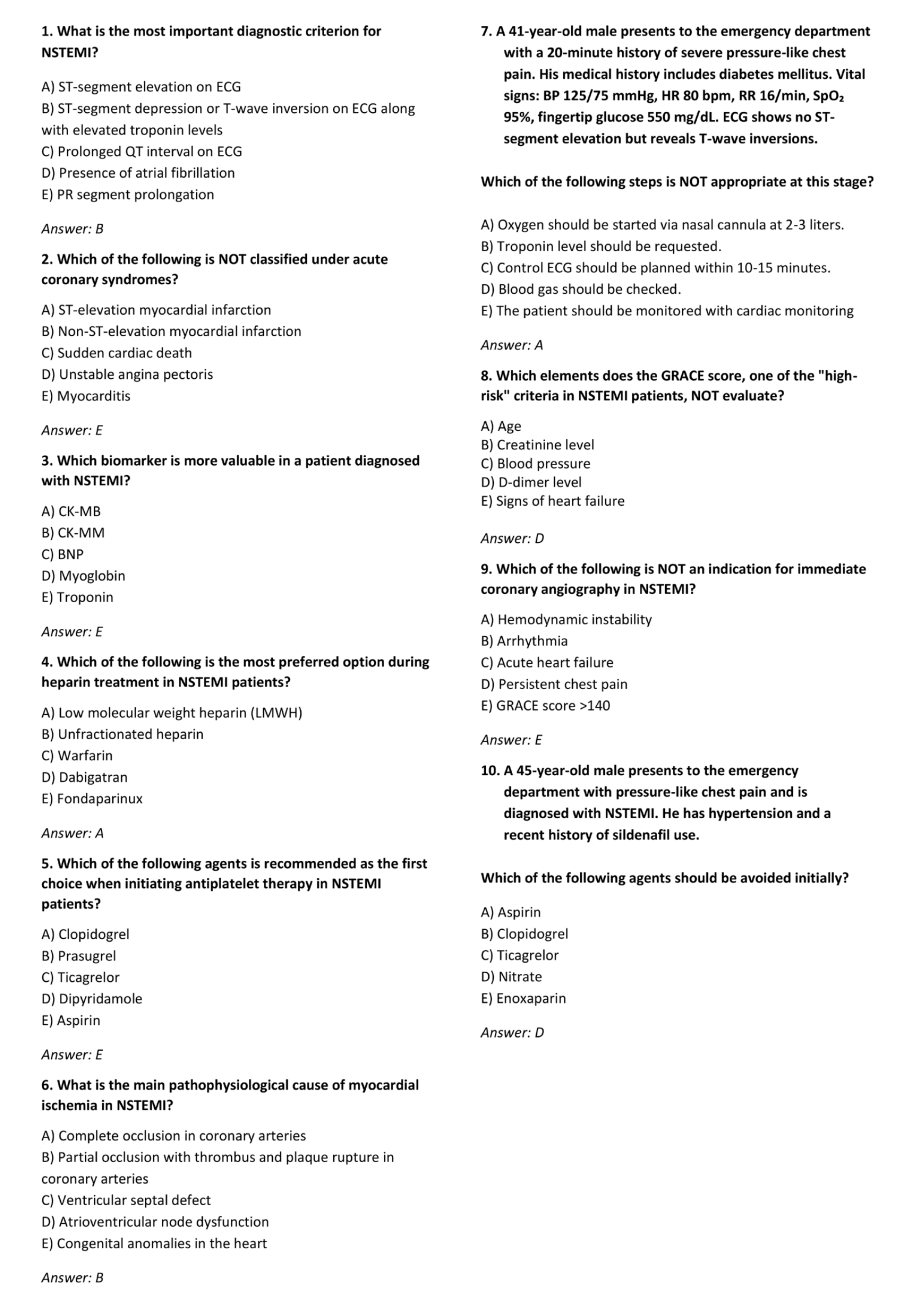
Survey Questions Used in the Study

 Data were collected using a questionnaire developed by the researchers based on a literature review. The questionnaire included demographic questions as well as ten multiple-choice items on NSTEMI, created in accordance with the 2023 European Society of Cardiology (ESC) guidelines (Figure 1). The items were reviewed by three independent emergency medicine specialists for content validity. Because each question assessed a distinct clinical concept, internal-consistency testing (Cronbach’s α) was not applicable. The same questions were also presented to two AI applications (ChatGPT 4.0 and Gemini 2.5), and their responses were independently analyzed. Both models were accessed via their official web interfaces on April 20, 2025, using an identical standardized prompt (“Answer as an experienced emergency physician according to the 2023 ESC NSTEMI guideline; select the single best option”). Temperature was set to 0 and web access disabled to ensure deterministic output. Each query was executed once, and AI-generated free-text responses were mapped to the corresponding MCQ option by two blinded researchers, with discrepancies resolved by consensus. This procedure minimized subjectivity and enhanced reproducibility.

 The questionnaire was prepared using the Google Forms platform and disseminated through professional networks including national emergency medicine society mailing lists, hospital emergency department email lists, and closed messaging groups (WhatsApp and Telegram) maintained by these organizations. Because these platforms do not provide access statistics, the exact number of physicians who received the invitation could not be determined, and a precise response rate or non-responder analysis could not be performed. Data were collected through an unproctored online survey, which limited control over participants’ environment and raised the possibility of external assistance or collaboration during completion. Although this approach enabled nationwide participation, it represents a convenience sample and may introduce selection, participation, and procedural bias. Informed consent was obtained electronically from all participants. Duplicate or incomplete submissions were excluded during data cleaning. As the survey platform did not record completion time, potential external consultation or unrealistic response behavior could not be formally audited.

 Ethical approval for the study was obtained from the Health Sciences Ethics Committee of the Faculty of Medicine at Manisa Celal Bayar University (Decision No: 12.05.2025/20.478.486/2887). Participation was voluntary, and it was assured that all data would be kept confidential and used solely for scientific purposes.

###  Statistical Analysis

 All statistical analyses were performed using SPSS version 26.0. Descriptive statistics for categorical variables were presented as frequencies and percentages, while continuous variables were reported using mean, standard deviation, median, minimum, and maximum values. The normality of continuous variables was assessed using the Kolmogorov–Smirnov test. For normally distributed continuous variables, independent samples t-tests or one-way ANOVA with Bonferroni correction were used for subgroup comparisons (title, experience, workplace). For contextual comparison, physicians’ mean scores were descriptively contrasted with the AI models’ fixed scores (9/10). However, because AI results are deterministic and lack sampling variance, no formal inferential test was applied to this comparison, and Cohen’s d values were reported only as descriptive indicators of magnitude rather than inferential effect sizes. A *P* value < 0.05 was considered statistically significant for subgroup analyses. For independent two-group comparisons of normally distributed continuous variables, the independent samples t-test was used; for comparisons involving more than two groups, one-way ANOVA was applied. In cases of variance heterogeneity, the Welch test was conducted. Depending on the assumption of homogeneity, Bonferroni or Tamhane’s post hoc tests were used. For categorical variables, chi-square tests were applied to assess differences between two independent groups. A *P* value of less than 0.05 was considered statistically significant for all analyses.

## Results

 A total of 1,106 physicians participated in the study, with a mean age of 27.9 ± 4.6 years (range: 23–60); 55.9% were female. Most participants (94.21%) graduated from public universities. In terms of workplace, 70.5% worked in state hospitals, 19.3% in training and research hospitals, 6.3% in university hospitals, and 3.8% in private hospitals. General practitioners made up the majority (74.68%), followed by residents (18.35%) and specialists (6.96%). Regarding professional experience, 47.11% had 0–1 year, 37.79% had 1–5 years, 8.86% had 5–10 years, and 6.24% had ≥ 10 years of experience. Among residents, seniority was distributed as follows: 0–1 year (28.6%), 1–2 years (22.2%), 2–3 years (28.1%), and 3–4 years (20.9%). These data are presented in Table 1.

**Table 1:** Demographic and Professional Characteristics of the Participants

**Category**	**n (%) or Mean±SD (Min–Max)**
Age (years), Mean ± SD (Min–Max)	27.9 ± 4.6 (23–60)
Gender, n (%)	
Male	488 (44.1)
Female	618 (55.9)
Graduation, n (%)	
Public university	1042 (94.21)
Private university	64 (5.79)
Workplace, n (%)	
State hospital	780 (70.5)
Training & research hospital	214 (19.3)
University hospital	70 (6.3)
Private hospital	42 (3.8)
Professional title, n (%)	
General practitioner	826 (74.68)
Resident	203 (18.35)
Specialist	77 (6.96)
Years of experience, n (%)	
0–1 year	521 (47.11)
1–5 years	418 (37.79)
5–10 years	98 (8.86)
≥ 10 years	69 (6.24)
Residency year, n (%)	
0–1 year	58 (28.6)
1–2 years	45 (22.2)
2–3 years	57 (28.1)
3–4 years	43 (20.9)

SD: Standard deviation; Mean: Average.

 The participating physicians were asked 10 multiple-choice questions. The overall mean number of correct answers among physicians was 7.62 ± 1.32. The same questions were presented to two AI applications, ChatGPT and Gemini, both AI models answered nine of ten questions correctly. ChatGPT provided an incorrect response to item 4 (initial management decision), whereas Gemini failed on item 7 (oxygen therapy indication), highlighting subtle differences in reasoning consistency.

 Analysis based on professional titles revealed that the mean number of correct answers was 7.48 ± 1.3 for general practitioners, 7.98 ± 1.1 for residents, and 8.19 ± 1.3 for specialists. According to the Welch test and Tamhane’s post hoc analysis, the scores of general practitioners were significantly lower than those of both residents and specialists (*P* < 0.001). No significant difference was observed between residents and specialists (Table 2).

**Table 2:** Mean Percentage of Correct Answers by Physician Title

**Title**	**n (%)**	**Mean±SD**	***P *** **value**
Resident	203 (18.3)	7.98 ± 1.1	< 0.001^*^
General practitioner	826 (74.6)	7.48 ± 1.3	
Specialist	77 (6.9)	8.19 ± 1.3	

^*^Welch’s test was performed, followed by Tamhane’s post-hoc test. SD: Standard Deviation, Mean: Average.

 According to the results of one-sample t-tests comparing the AI applications’ mean score of 9 with the average scores of the professional title groups, all three physician groups scored significantly lower than the AI (*P* < 0.001). When effect sizes were evaluated using Cohen’s d, the difference was very large between general practitioners and AI (d = –1.17), large between residents and AI (d = –0.93), and moderate between specialists and AI (d = –0.62) (Table 3).

**Table 3:** Comparison of Mean Percentage of Correct Answers Between AI and Physician Groups

**Title**	**n (%)**	**Mean±SD**	**t**	**df**	***P *** value^*^	**Cohen’s d**
Resident	203 (18.3)	7.98 ± 1.1	-12.469	202	< 0.001	-0.93
General practitioner	826 (74.6)	7.48 ± 1.3	-33.020	825	< 0.001	-1.17
Specialist	77 (6.9)	8.19 ± 1.3	-5.360	76	< 0.001	-0.62

d: Cohen’s d (effect size); SD: standard deviation; Mean: average; df: degrees of freedom. ^*^One-sample t-test

 In the one-sample t-tests comparing the AI application’s performance with physician groups categorized by years of professional experience, all groups had significantly lower mean scores than the AI (*P* < 0.001). According to effect size analysis, the difference was very large for physicians with 0–1 year (d = –1.17) and 1–5 years (d = –1.05) of experience. In the 5–10 years group, the difference was large (d = –0.74), and in the ≥ 10 years group, it was also large (d = –0.91) (Table 4).

**Table 4:** Comparison of Mean Percentage of Correct Answers Between AI and Physician Groups by Years of Experience

**Professional experience**	**n (%)**	**Mean±SD**	**t**	**df**	P*	**Cohen’s d**
0–1 year	521 (47.1)	7.48 ± 1.29	-26.80	520	< 0.001	-1.17
1–5 years	418 (37.8)	7.67 ± 1.3	-20.54	417	< 0.001	-1.05
5–10 years	98 (8.9)	8.10 ± 1.22	-7.27	97	< 0.001	-0.74
≥ 10 years	69 (6.2)	7.68 ± 1.45	-7.55	68	< 0.001	-0.91

d: Cohen’s d (effect size); SD: standard deviation; Mean: average; df: degrees of freedom. ^*^One-sample t-test Cohen’s d values are presented as descriptive indicators of magnitude of difference, not as inferential statistics, given the deterministic nature of AI scores.

 A one-way analysis of variance (ANOVA) was conducted to identify differences in the number of correct answers based on years of professional experience. According to Bonferroni multiple comparison tests, a significant difference was observed between physicians with 0–1 year and those with 5–10 years of experience (*P *< 0.001), as well as between those with 1–5 years and 5–10 years of experience (*P *= 0.020). No significant differences were found in the other group pairs (Table 5).

**Table 5:** Comparison of Mean Percentage of Correct Answers by Years of Professional Experience

**Years of experience**	**Comparison**	**Mean difference**	**Standard Error**	***P***	**95% Confidence interval (lower–upper)**
0–1 year	1–5 year	-0.194	0.086	0.147	-0.42 – 0.03
	5–10 years	-0.626^*^	0.144	**<0.001**	-1.01 – -0.24
	≥ 10 years	-0.205	0.168	1.000	-0.65 – 0.24
1–5 years	0–1 year	0.194	0.086	0.147	-0.03 – 0.42
	5–10 years	-0.432^*^	0.147	**0.020**	-0.82 – -0.04
	≥ 10 years	-0.011	0.170	1.000	-0.46 – 0.44
5–10 years	0–1 year	0.626^*^	0.144	**<0.001**	0.24 – 1.01
	1–5 years	0.432^*^	0.147	**0.020**	0.04 – 0.82
	≥ 10 years	0.421	0.206	0.248	-0.12 – 0.97
≥ 10 years	0–1 year	0.205	0.168	1.000	-0.24 – 0.65
	1–5 years	0.011	0.170	1.000	-0.44 – 0.46
	5–10 years	-0.421	0.206	0.248	-0.97 – 0.12

^*^Bonferroni test, *P* < 0.05 indicates a statistically significant difference.

 In the analysis based on the type of university from which the participants graduated, no statistically significant difference was found between those who graduated from public and private universities. However, according to the Welch test performed by hospital type, physicians working in training and research hospitals had a significantly higher mean number of correct answers (7.88 ± 1.17), while those working in state hospitals had the lowest mean score (7.54 ± 1.31) (*P* = 0.004).

**Figure 2:**
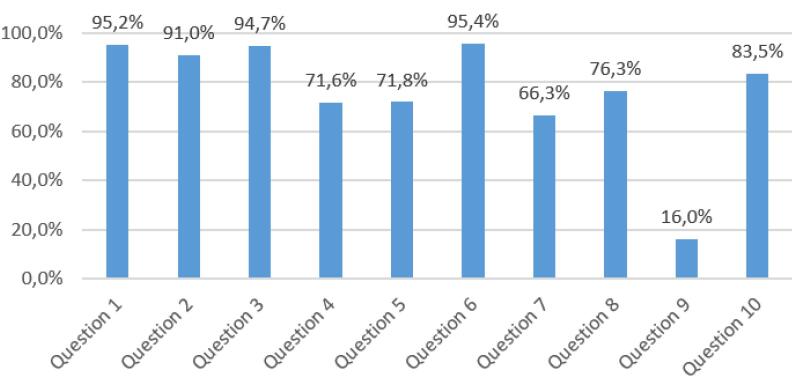
Correct Response Rates for the Questions

 Item-level comparisons between AI models and physicians are presented in Figure 2, illustrating concordance and discordance across the ten NSTEMI questions. When evaluating the correct answer rates for the questions, questions 1, 2, 3, and 6 were answered correctly by over 90% of all experience groups. The lowest correct response rate was 16% for question 9, which ChatGPT answered incorrectly. The only question Gemini answered incorrectly was question 7, which had a correct response rate of 66.3% (Figure 2).

## Discussion

 ChatGPT and Gemini AI models significantly outperformed general practitioners, residents, and specialists on ten NSTEMI-related multiple-choice questions, correctly answering nine out of ten compared to the physicians’ average of 7.62 ± 1.32. While formal inferential testing was not applied due to the deterministic nature of AI scores, descriptive comparisons and effect size estimates suggested substantial differences, particularly among less experienced physicians. However, this higher accuracy in multiple-choice questions does not equate to clinical diagnostic superiority, as no real-case simulations or workflow evaluations were performed. Likewise, multiple-choice accuracy reflects guideline knowledge rather than complex diagnostic reasoning, which involves contextual judgment, uncertainty management, and dynamic patient interaction.

 The high accuracy of AI systems highlights their potential as effective support tools in knowledge-driven tasks, particularly in time-critical settings like emergency departments.^7,8^ However, the human factor remains crucial. Johnson et alreported that AI alone achieved 92% diagnostic accuracy, which dropped to 76% when used alongside physicians, suggesting possible misinterpretation of AI outputs by clinicians.^9^ This suggests that while AI can be a powerful tool, its integration into clinical practice requires careful consideration of how clinicians interpret and utilize AI-generated information.

 Recent studies have tested the clinical performance of LLMs such as ChatGPT-4 and Gemini across various scenarios. Gilson et al^10^ reported an initial diagnostic accuracy of 54.6% for ChatGPT-4, significantly higher than other models. Another study showed that ChatGPT scored above the 60% passing mark on the multiple-choice questions of the European Core Cardiology Examination (EECC), demonstrating strong performance.^11^ In a 50-question study, ChatGPT-4 achieved an 82.1% accuracy rate, while physicians scored 83.7%, with no statistically significant difference between them.^12^ In our study, AI models reached a 90% accuracy rate, which may be attributed to the more specific focus on NSTEMI. This suggests that LLMs may perform better in targeted clinical domains.

 A significant association was observed between physicians’ professional experience and their performance. The group with 5–10 years of experience demonstrated the highest success, with a moderate effect size observed between this group and AI. These findings suggest that clinical decision-making skills improve up to a certain experience threshold, after which they plateau. However, AI models surpassed this threshold, outperforming even the most experienced physicians.

 Institutional settings also influenced knowledge levels. Physicians in training and research hospitals demonstrated higher accuracy rates compared to those in state hospitals. This supports the role of academic environments in promoting guideline-based knowledge updates and highlights the contribution of organizational learning culture to knowledge enhancement.

 Questions covering basic knowledge such as diagnosis, biomarkers, and pathophysiology (e.g. questions 1, 3, and 6) showed high accuracy ( > 90%). However, accuracy declined for items requiring clinical reasoning, treatment decisions, and guideline interpretation. Notably, question 9 (‘urgent coronary angiography indication’) had only a 16% correct response rate, with ChatGPT also answering incorrectly. Question 7 (‘oxygen therapy necessity’) had a 66.3% correct rate, where Gemini failed. These results highlight the need for improved clinical reasoning and guideline interpretation in both physicians and AI. Supporting this, Li et al. demonstrated frequent failures of LLMs in guideline-based clinical decisions.^13^ Similarly, Williams et al reported that LLMs lagged behind clinicians in triage, diagnosis, and treatment guideline interpretation using the MIMIC-IV dataset.^14^ These item-level errors indicate that AI models may struggle with context-dependent or ambiguous clinical scenarios, emphasizing the continued need for human oversight in clinical decision-making.

 The generally consistent and superior performance of AI models indicates their potential as supportive tools in medical education and continuous professional development. With their capacity for large-scale data analysis and rapid processing of up-to-date guideline information, AI systems can provide significant contributions to clinical decision support mechanisms for physicians.^15^ However, these findings should not be extrapolated to clinical workflow integration or policy-level implementation, as the present study assessed only knowledge-based question performance without real-world decision-making or safety evaluation.

 This study has limitations. Recruitment through professional mailing lists and closed messaging groups prevented determination of the total number of physicians who received the invitation; therefore, the sample should be considered convenience-based. Because participation occurred in an unproctored online environment, participation bias and lack of control over completion settings may have influenced results. In addition, potential variability in AI responses due to prompt sensitivity and the deterministic nature of model outputs limit internal validity and the interpretability of statistical comparisons. The questionnaire included multiple-choice questions only, which assess guideline-based knowledge rather than complex clinical reasoning or real-case decision-making. Limited information on AI model versions and training data, along with their rapidly evolving nature, may affect reproducibility over time. Finally, the study focused solely on NSTEMI and did not include patient-level outcomes; results should therefore be interpreted as reflecting knowledge-based rather than clinical outcome performance.

## Conclusion

 This study shows that ChatGPT and Gemini outperform emergency physicians in NSTEMI clinical questions. These results underscore AI’s potential to enhance medical education, clinical decision support, and ultimately, patient care through faster information access and improved diagnostic accuracy.

 However, these findings should be interpreted in light of the study’s methodological constraints, including its online, non-clinical design and the rapidly evolving nature of AI models. The physician’s critical role remains essential, especially in complex or uncertain clinical scenarios. Future research should focus on integrating AI into clinical workflows, optimizing interaction and collaboration between physicians and AI, testing AI performance in different clinical scenarios, and ensuring that the integration is safe, ethical and effective. Such integration promises to usher in a new era in medicine, significantly improving patient outcomes and optimizing healthcare delivery.
